# Applications of artificial intelligence in personalized nutrition interventions

**DOI:** 10.3389/fnut.2026.1974199

**Published:** 2026-09-28

**Authors:** Yahui Zhang, Meijing Feng, Qiuyan Xue, Rui Ma, Fangming Liu

**Affiliations:** Department of Health Management, Aerospace Center Hospital, Beijing, China

**Keywords:** artificial intelligence, continuous glucose monitoring, digital twin, gut microbiome, machine learning, multi-omics, nutrigenomics, personalized nutrition

## Abstract

Unhealthy dietary patterns remain a major modifiable contributor to the global burden of obesity, type 2 diabetes mellitus (T2DM), cardiovascular disease, and cancer. However, individuals can respond markedly differently to identical foods, limiting the effectiveness of one-size-fits-all dietary guidance. This review examines the application of artificial intelligence (AI) to personalized nutrition interventions across three interconnected layers. At the biological level, inter-individual variability can be characterized by three mechanistic dimensions: nutrigenomic variation, which influences nutrient metabolism and disease susceptibility; metabolomic biomarkers, which objectively capture dietary exposure and early metabolic alterations; and the gut microbiome, which bidirectionally mediates diet–host interactions. At the technological level, AI integrates these dimensions into multimodal digital profiles. Machine learning models predict individual postprandial glycemic and lipemic responses, with reported correlations of up to r = 0.77 in large cohorts; deep learning and computer vision automate dietary assessment; natural language processing and large language models (LLMs) support nutrition counseling; knowledge graphs enable constraint-aware food recommendations; and digital-twin and adaptive closed-loop systems incorporating continuous glucose monitoring and wearable-device data support real-time nutritional interventions. However, the strength of clinical evidence varies across disease domains. Glycemic management in T2DM and prediabetes currently has the most mature randomized controlled trial evidence: an AI-enabled personalized intervention combining algorithmic dietary personalization with CGM-linked feedback improved glycemic endpoints compared with a Mediterranean diet, while a fully automated AI-delivered diabetes prevention program achieved outcomes non-inferior to those of human coaching. In obesity, cardiovascular disease, oncology, and gastrointestinal disorders, the evidence remains mixed or preliminary. Clinical translation also faces substantial challenges, including biased and inconsistently standardized dietary and multi-omics data; the underrepresentation of non-European populations, which limits model generalizability and raises concerns about health equity; limited model explainability; privacy and data-governance challenges; immature regulatory pathways for adaptive software as a medical device; and specific risks associated with LLMs, such as hallucinated or guideline-discordant dietary advice. AI-enabled personalized nutrition is therefore progressing from proof of concept toward clinical translation. Realizing its potential equitably will require multi-ethnic nutrition–omics cohorts, privacy-preserving multicenter collaboration, dietitian-led human–AI collaboration, and lifecycle safety governance for generative models.

## 1 Introduction

John McCarthy coined the term “artificial intelligence (AI)” at the Dartmouth Conference in 1956 (1), although its conceptual foundations can be traced back to Alan Turing's work on machines capable of emulating human intelligence and the development of the Turing test. With advances in computing power, AI has become increasingly integrated into daily life, and medicine has been particularly receptive to its application. AI is now widely used across a range of medical specialties, including radiology, pathology, ophthalmology, orthopedics, and obstetrics and gynecology (2–6), where it has improved the accuracy and efficiency of diagnosis and treatment.

Unhealthy dietary patterns are well-established drivers of obesity and diet-related diseases, including type 2 diabetes, cardiovascular disease (CVD), stroke, and cancer (7). Within the framework of precision medicine, multi-omics technologies, including nutrigenetics, nutrigenomics, and gut microbiome research, have reshaped nutritional research and made precision nutrition interventions an important focus of academic investigation (8–10). By integrating an individual's nutritional and metabolic profiles, genetic characteristics, gut microbial composition, and lifestyle factors, this approach enables the development of multidimensional dietary intervention strategies aimed at preventing disease, improving health outcomes, and reducing associated economic burdens (11). Throughout this review, we use *personalized nutrition* (PN) as the primary term to describe the tailoring of dietary guidance to individual characteristics, while *precision nutrition* is used interchangeably where appropriate.

AI is already transforming the implementation of PN. Multimodal data-analysis frameworks, including ensemble learning and deep neural networks, can identify complex patterns within large-scale nutritional and dietary intake datasets (12) and provide quantitative predictions of individual responses. In the PREDICT 1 cohort (*n* = 1,002), a machine learning (ML) model integrating dietary, clinical, and gut microbiome features predicted individual postprandial glycemic responses with a correlation of r = 0.77 and postprandial triglyceride (TG) responses with r = 0.47 (13). As reported in a systematic review, random forest algorithms can jointly analyze genetic and dietary data to characterize complex gene–diet interactions (14). Food recognition technologies have progressed in parallel, with deep learning (DL) evolving from image classification toward direct nutrient estimation. The Nutrition5k dataset, which contains approximately 5,000 real-world dishes with ingredient-level nutritional annotations, demonstrated that DL models can directly estimate nutrient content from food images, while video-based portion-size estimation achieved lower errors than human visual assessment (15). Systematic reviews of this field have documented the rapid development of convolutional and deep neural networks for food image classification and nutrient identification, while also highlighting persistent limitations related to dataset size and annotation quality (16). Overall, AI-driven multimodal data analytics provides a systematic and quantitative foundation for applying PN to disease prevention, diagnosis, monitoring, and treatment.

Two recent reviews complement the scope of the present work: one surveys AI approaches to PN together with applications in food manufacturing (17), while the other focuses specifically on large language models in clinical nutrition (18). The present review differs from both in its organization and emphasis. It anchors AI applications in the biological data layers described above, evaluates clinical evidence across individual disease domains with particular attention to randomized controlled trials (RCTs), effect sizes, and translational readiness, and considers governance as an issue spanning the entire implementation pipeline. Accordingly, this review examines, through the three lenses of genomics, metabolomics, and the gut microbiome, how individual physiological and genetic characteristics shape PN interventions. Building on this biological foundation, we further examine how contemporary AI approaches, including ML, DL, and knowledge graphs (KGs), are being applied, evaluate the current role of PN in disease prevention, and summarize the challenges associated with using AI to develop individualized nutrition plans. Figure 1 illustrates the overall conceptual framework of this review.

**Figure 1:**
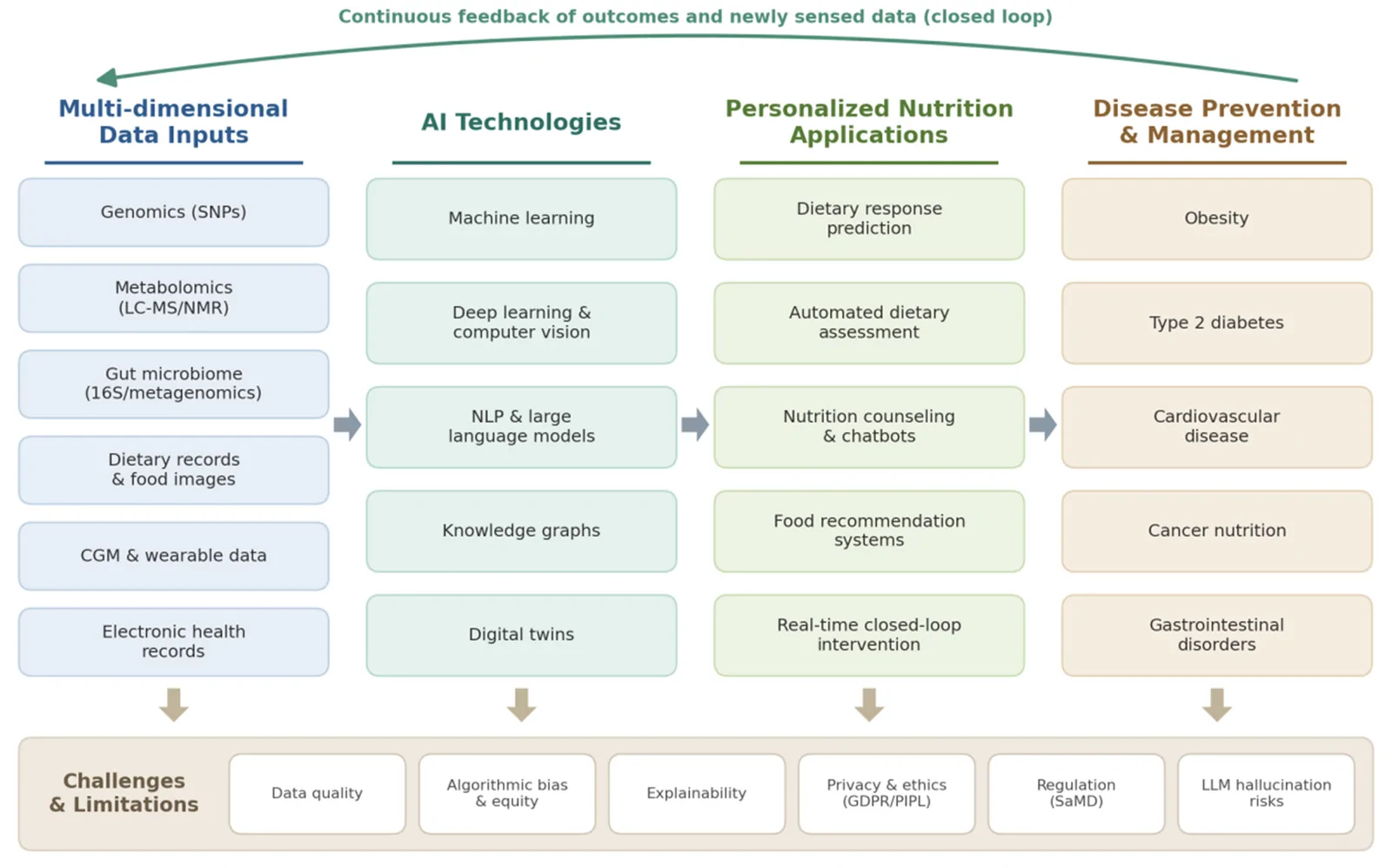
Overall framework of artificial intelligence (AI) applications in personalized nutrition interventions. Multidimensional data inputs, including genomics, metabolomics, gut microbiome profiles, dietary records and food images, continuous glucose monitoring (CGM) and wearable-device data, and electronic health records, are integrated and processed using AI technologies, including machine learning, deep learning and computer vision, natural language processing (NLP) and large language models, knowledge graphs, and digital-twin or adaptive closed-loop systems. These technologies support a range of personalized nutrition applications, from dietary response prediction and automated dietary assessment to nutrition counseling chatbots, food recommendation systems, and real-time closed-loop interventions, with the ultimate goal of preventing and managing obesity, type 2 diabetes, cardiovascular disease, cancer, and gastrointestinal disorders. The curved return arrow indicates the continuous feedback of intervention outcomes and newly acquired data into the data layer, thereby closing the loop. The bottom band summarizes the cross-cutting challenges spanning the entire pipeline, including data quality, algorithmic bias and equity, explainability, privacy and ethics (GDPR/PIPL), regulation of software as a medical device (SaMD), and the risk of hallucinations in large language models.

### 1.1 Literature search strategy

This article is a narrative review; therefore, no systematic screening protocol, such as PRISMA, was applied. Relevant literature was identified through PubMed/MEDLINE and Google Scholar, supplemented by publisher full-text platforms. We focused primarily on studies published between January 2015 and September 2026, although seminal foundational studies were not subject to this date restriction. Search terms combined (“artificial intelligence” OR “machine learning” OR “deep learning” OR “large language model” OR “knowledge graph” OR “digital twin”) with (“personalized nutrition” OR “precision nutrition” OR “nutrigenomics” OR “metabolomics” OR “gut microbiota” OR “dietary assessment” OR “nutritional intervention”). Disease-specific sections additionally incorporated the terms (“obesity” OR “type 2 diabetes” OR “cardiovascular disease” OR “cancer nutrition” OR “gastrointestinal”). Priority was given to randomized controlled trials, systematic reviews and meta-analyses, prospective cohort studies, and major original studies relevant to the clinical translation of AI-enabled personalized nutrition. Foundational studies were included where necessary to establish key biological or technological concepts. Newly included references were cross-verified across databases for consistency in title, authorship, journal, publication year, and DOI. Because no formal systematic evidence-grading or risk-of-bias framework was applied, comparisons of evidence maturity across AI applications and disease domains should be interpreted as a structured qualitative synthesis rather than formal comparative evidence grading.

## 2 Exploration of PN at the genomic level

High-throughput gene sequencing and multi-omics analytical techniques (19, 20) have enabled researchers to characterize how genetic variation contributes to interindividual differences in nutrient metabolism, dietary response, and susceptibility to diet-related diseases. Genome-wide and candidate-gene studies have identified numerous diet-relevant variants, including loci such as ADRB2, TCF7L2, FABP2, FTO, APOE, and MTHFR, which have been associated with glycemic regulation, obesity, lipid metabolism, and nutrient handling (21–24). Additional studies have linked FTO variants to energy intake and obesity-related phenotypes, FGF21-region variation to macronutrient intake, and APOE and MTHFR genotypes to disease susceptibility and lifestyle-related phenotypes (27–36); related genetic heterogeneity has also been highlighted in precision hypertension (37). However, the predictive and interventional value of individual genetic variants remains limited. In the Diabetes Prevention Program, lifestyle intervention reduced progression to diabetes regardless of TCF7L2 risk-allele status, with no significant genotype–intervention interaction (25). Moreover, established clinical risk factors, including family history, age, sex, BMI, and fasting glucose, can outperform individual genetic variants or polygenic scores in predicting type 2 diabetes (26). These observations suggest that genomic information is most informative when considered as one component of a broader multidimensional profile rather than as a stand-alone basis for dietary prescription.

Genomic and epigenomic information therefore provides a relatively stable layer of biological variation that can complement more dynamic metabolic, microbial, and behavioral data in PN. Gene–diet interactions remain an important mechanistic basis of personalized dietary responses (20), while epigenetic measures such as DNA methylation may capture additional biological variation beyond static genotype (38). Intervention studies further suggest that personalized dietary advice can improve dietary behavior: in the Food4Me study, individualized nutrition advice was associated with greater improvements in diet quality than generalized recommendations (39, 40). From the perspective of AI-assisted PN, the principal value of genomic data lies in their integration with other omics, clinical, and lifestyle variables, allowing algorithms to identify complex interaction patterns that are unlikely to be captured by single genetic markers alone.

## 3 Metabolomics and PN

Metabolomics provides a dynamic complement to genomic information by characterizing metabolic responses to dietary exposure. By profiling endogenous and exogenous small-molecule metabolites in biological specimens, metabolomic analyses can capture dietary intake, bioactive compounds, and diet-induced alterations in host metabolic pathways (41, 42). These metabolic signatures can serve as objective dietary and metabolic biomarkers and can be integrated with other multi-omics datasets to support predictive modeling and PN (43, 44). NMR- and mass spectrometry-based platforms, using targeted or untargeted approaches, enable the identification and validation of such metabolic signatures (45–47). Compared with relatively static genomic information, metabolomic profiles provide a more dynamic readout of an individual's current physiological response to diet.

Metabolic signatures associated with glucose regulation, lipid metabolism, amino-acid metabolism, and related pathways have been linked to insulin resistance, T2DM, obesity, and other metabolic disorders (48–54). For example, serum metabolic profiling has identified diabetes-associated biomarkers (49, 50), while lipid, acylcarnitine, and branched-chain amino-acid signatures provide additional information on metabolic dysfunction (51, 52, 54). Lipid- and fatty-acid-related evidence further illustrates this metabolic heterogeneity, with associations varying across lipoprotein fractions, dietary fatty-acid composition, fatty-acid chain length, tissue context, and disease state (55–60). However, these metabolites are highly intercorrelated and are influenced by diet, disease state, host genetics, microbiota, and other environmental factors. Their value for PN therefore lies less in isolated biomarkers than in the multivariate patterns they collectively provide. From the perspective of AI-assisted PN, high-dimensional metabolomic profiles can be integrated with genomic, microbiome, clinical, and behavioral data to support individualized risk stratification, dietary-response prediction, and longitudinal monitoring.

## 4 Gut microbiome and PN

The gut microbiome represents another major source of interindividual variability relevant to PN. Its composition and functional capacity are shaped by habitual diet and, in turn, influence nutrient transformation, metabolite production, energy homeostasis, and host metabolic responses (61–65, 74). Microbiome alterations have been associated with obesity, T2DM, inflammatory disorders, and other chronic diseases (66–70), supporting the use of microbial features as potential biomarkers for individualized nutritional assessment. Microbiome-based biomarkers and microbiota-targeted interventions have also been explored in cancer and cardiometabolic diseases (71–73).

Importantly for PN, diet–microbiome interactions are dynamic and bidirectional. Dietary patterns such as the Mediterranean diet can modify microbial composition and microbial metabolite production, including short-chain fatty acids, whereas less favorable dietary patterns may be associated with reduced microbial diversity and pro-inflammatory metabolic features (75–80, 87, 88). Beyond microbiome composition, adherence to Mediterranean dietary patterns has been associated with favorable cardiovascular, weight-related, glycemic and metabolic, and cancer-related outcomes (81–86). However, substantial interpersonal variability in baseline microbiome composition means that identical dietary exposures may produce different microbial and metabolic responses across individuals.

The microbiome is therefore particularly relevant to AI-assisted PN because it generates high-dimensional, context-dependent data that can complement genomic, metabolomic, dietary, and physiological information. Evidence from intervention studies, humanized models, multi-omics analyses, and genetic causal-inference approaches further supports biological links between microbiome variation and host phenotypes (89, 90). Rather than using individual taxa as deterministic dietary markers, subsequent AI approaches can integrate microbial community and functional features with other data layers to improve individualized response prediction.

Together, genomics, metabolomics, and the gut microbiome provide complementary but individually incomplete representations of nutritional heterogeneity. Their principal value for AI-assisted PN lies in multimodal integration, through which complex interactions among relatively static predisposition, dynamic metabolic state, and environmentally responsive microbial features can be jointly modeled to predict individualized dietary responses.

## 5 AI-assisted PN

### 5.1 Overview: from single-dimension data to multimodal digital profiling

PN integrates multidimensional individual-level information with targeted behavioral interventions to provide tailored dietary recommendations and promote health optimization (91). In parallel with this transition, AI has increasingly been incorporated into clinical nutrition by enabling the analysis of complex datasets, interpretation of medical images, and generation of individualized intervention strategies. AI-powered devices and applications further facilitate dietary monitoring, provide real-time feedback, and support behavioral modification toward healthier eating patterns (92, 93). Importantly, the application of AI in nutrition extends beyond the analysis of individual data types. By integrating diverse biological layers—including genomics, metabolomics, and microbiomics—with behavioral information collected from wearable devices, AI-based systems can construct comprehensive digital representations of individual nutritional states. Some of these systems have been described as digital twins; however, continuously updated personalized digital profiles should not be considered digital twins unless they maintain a dynamic individualized computational representation that is repeatedly synchronized with real-world data and used to simulate, predict, or optimize future states or interventions.

AI technologies encompass a spectrum of approaches, ranging from task-specific algorithms to broader cross-domain systems. The major technical foundations include ML, DL, natural language processing (NLP), and computer vision (CV) (94, 95). These technologies contribute to different stages of the PN workflow: ML enables the integration of heterogeneous omics and clinical variables to predict individual responses to dietary interventions; DL and CV facilitate automated food recognition, dietary assessment, and quantification; NLP, including emerging large language models (LLMs), enhances human-AI interaction and information processing; while KG-based reasoning and digital twin frameworks support the transformation of predictive outputs into dynamic and actionable dietary recommendations.

Early applications of AI-assisted nutrition primarily relied on single-dimensional datasets, such as dietary records, circulating biomarkers, or genetic information. In contrast, contemporary approaches increasingly emphasize multimodal integration, combining gut microbiome profiles, continuous glucose monitoring (CGM) data, metabolomic signatures, and behavioral information within unified predictive frameworks. This transition represents more than a simple expansion of data volume; it has fundamentally reshaped our understanding of nutritional responses by demonstrating that interindividual variability in metabolic responses to identical dietary exposures may exceed the variability attributable to the dietary intervention itself. Because the studies discussed below evaluate AI-enabled PN at different stages of translation, we distinguish five complementary levels of evidence throughout this review: (1) technical validity, including model correlation, discrimination, and prediction-error metrics; (2) external validity, reflecting reproducibility in independent populations or settings; (3) clinical efficacy, assessed by changes in prespecified clinical or metabolic endpoints in prospective intervention studies; (4) implementation effectiveness, including feasibility, adherence, engagement, and performance under real-world delivery conditions; and (5) patient-important outcomes, such as disease incidence, major clinical events, quality of life, or mortality. These levels should not be interpreted interchangeably: strong predictive performance does not by itself establish clinical efficacy or implementation effectiveness, and improvement in surrogate endpoints does not necessarily demonstrate long-term patient benefit. This framework is used below to interpret the translational maturity of different AI-enabled PN applications.

### 5.2 ML for multi-omics integration and dietary response prediction

One of the most influential applications of ML in PN is the integration of multi-omics and clinical data to predict individual postprandial responses. A landmark study by Zeevi et al. (96) continuously monitored 800 individuals using CGM for 1 week and recorded 46,898 meals, revealing substantial interindividual variability in postprandial glycemic responses (PPGRs) to identical foods. The authors developed a gradient-boosting regression model incorporating blood parameters, dietary habits, anthropometric characteristics, physical activity, and gut microbiome profiles. The model successfully predicted individual PPGRs in an independent validation cohort of 100 participants. Moreover, a short-term dietary intervention based on algorithmically generated “good” and “bad” diets effectively reduced postprandial glucose levels. This study established a central principle of the field: because dietary responses are highly individualized, accurate prediction requires person-specific characteristics rather than food-related variables alone.

Subsequent studies extended this framework in three major directions. First, researchers further examined the contribution of the gut microbiome to individual dietary responses. In a randomized crossover trial involving 20 healthy participants who consumed either sourdough whole-grain bread or industrially produced white bread for 1 week each, Korem et al. (97) observed no significant average difference in clinical outcomes between the two bread types. However, individual glycemic responses varied markedly and, importantly, could be predicted from baseline microbiome profiles alone. These findings suggested that even the direction of an individual's metabolic response to a specific food may differ according to personal biological characteristics. Second, the PREDICT program substantially expanded both the scale and multimodal scope of dietary response prediction. Berry et al. (13) profiled 1,002 twins and unrelated healthy adults in the United Kingdom, together with an independent validation cohort of 100 participants in the United States. Pronounced interindividual variability was observed in postprandial TG, glucose, and insulin responses, with coefficients of variation of 103%, 68%, and 59%, respectively. Individual-level factors, including the gut microbiome, explained a greater proportion of the variance in postprandial lipemia (7.1%) than meal macronutrient composition (3.6%), whereas genetic factors made relatively modest contributions, accounting for 9.5% of the variance in glucose responses and 0.8% in TG responses. An ML model integrating these multimodal variables predicted postprandial TG (r = 0.47) and glycemic (r = 0.77) responses. Complementary deep phenotyping of 1,098 PREDICT 1 participants by Asnicar et al. (98) identified 30 microbial species associated with healthy or unhealthy dietary patterns and cardiometabolic markers. Microbiome-derived features also contributed independently to the prediction of postprandial responses beyond calorie and nutrient intake. Third, research has increasingly progressed from response prediction toward personalized dietary intervention. In the 6-month PERSONAL RCT, an ML-derived personalized postprandial-targeting (PPT) diet produced greater improvements in glycemic control than a Mediterranean diet among adults with prediabetes, with benefits maintained at the 12-month follow-up [(99); detailed in Section 6.2]. A subsequent integrative analysis further showed that diet-induced changes in microbial richness and composition were associated with improvements in cardiometabolic outcomes. In addition, ML models based on dietary changes and baseline clinical characteristics were able to predict individual responses to the 6-month intervention (100).

Despite these advances, external validity remains a major challenge. Mendes-Soares et al. (101) evaluated the transferability of the Zeevi model, originally developed in an Israeli cohort, in 327 adults without diabetes recruited at the Mayo Clinic in the United States. Over 6 days of CGM monitoring, predicted and measured PPGRs remained significantly correlated (R = 0.62), outperforming models based solely on calorie content (R = 0.34) or carbohydrate content (R = 0.40). These findings provide encouraging evidence for cross-population applicability. Nevertheless, many existing models have been developed in relatively homogeneous cohorts, often with substantial representation of individuals of European ancestry. Although genetic factors explained only a modest proportion of response variability in PREDICT 1, their contribution may differ across ancestries, disease states, and environmental contexts. Whether current models can maintain predictive performance across more diverse populations, longer follow-up periods, and the complexity of free-living dietary behavior therefore remains an important unresolved issue, as discussed further in Section 7.

A further limitation is the heterogeneity of model-performance reporting across studies. Some studies primarily report correlation coefficients or discrimination metrics, whereas others provide prediction-error measures, calibration, or decision-curve analyses. RMSE, MAE, prediction intervals, calibration, and clinically meaningful classification performance are not consistently reported across the current literature, making direct comparison of model performance difficult. Accordingly, where available, we report complementary error, calibration, and clinical-performance metrics rather than relying solely on correlation coefficients or AUCs. Future studies should more consistently report discrimination, calibration, prediction error, uncertainty estimates, and clinically meaningful performance measures to facilitate external validation and clinical interpretation.

### 5.3 DL and CV for automated dietary assessment

Accurate dietary assessment remains a major data-acquisition bottleneck in PN because conventional self-reported food diaries and dietary recalls are time-consuming and susceptible to systematic reporting bias. DL and CV provide a potential solution by enabling food images to be translated directly into estimates of portion size and nutrient intake. Im2Calories, introduced by Myers et al. (102), was among the pioneering systems in this field. It combined deep convolutional neural networks for food recognition and segmentation with monocular depth estimation to infer food volume and, ultimately, caloric content from a single image. The system achieved a top-1 food-recognition accuracy of 79% on the Food-101 test set and demonstrated depth-based volume estimation using the NFood-3d dataset, which contained 1,050 depth images of replica-meal assemblies. However, end-to-end calorie estimation was not quantitatively evaluated.

Subsequent progress has been driven by improvements in both model architecture and dataset quality. Thames et al. (15) introduced Nutrition5k, a dataset comprising approximately 5,000 real-world dishes with overhead and side-view video recordings, ingredient-level weights, and high-precision nutritional annotations. Their baseline experiments demonstrated the feasibility of directly estimating nutrient content from food images and showed that video-based portion estimation could outperform human visual estimation. The study also highlighted an important limitation in the field: many publicly available food-image datasets contain relatively coarse or incomplete nutritional annotations, restricting their utility for accurate nutrient estimation. In a clinical setting, Rhyner et al. (103) compared the mobile phone-based GoCARB system, which integrates image recognition with food-volume reconstruction, with carbohydrate self-estimation by individuals with type 1 diabetes. The system produced significantly more accurate pre-meal carbohydrate estimates than participants' own assessments, indicating that CV-assisted dietary assessment may improve the quality of dietary information used for insulin-dose calculation.

Despite substantial methodological advances from manually annotated image regions to increasingly automated DL-based pipelines, several important limitations remain. A recent scoping review of image-based calorie-counting applications for people with diabetes identified automated food-volume and portion-size estimation as a persistent technical challenge. It also noted that available datasets remain geographically and culturally biased and that validation across diverse populations is limited, with most applications yet to progress beyond the research stage (104). Multi-view imaging, depth sensing, and three-dimensional reconstruction are increasingly used to improve portion-volume estimation, but rigorous clinical evaluations comparable to those conducted for systems such as GoCARB remain uncommon. Therefore, until these technical and validation gaps are addressed, CV-based dietary assessment is better positioned as a complementary tool to structured dietary reporting rather than a complete replacement for conventional assessment methods.

### 5.4 NLP and large language models for nutrition counseling

NLP provides the conversational interface for PN, initially through task-oriented chatbots designed to support behavior change and, more recently, through general-purpose LLMs. In a systematic review of nine studies, including four RCTs and five quasi-experimental studies, AI chatbots showed potential for improving physical activity. However, the short follow-up periods and small sample sizes precluded definitive conclusions regarding dietary outcomes or weight management (105). A subsequent meta-analysis of 19 trials reported small-to-moderate but significant improvements in diet quality and physical activity. Subgroup analyses further indicated that, for fruit and vegetable intake, text-based chatbots outperformed voice-based systems, while AI/NLP-driven chatbots performed better than non-AI/NLP variants (95).

LLMs such as ChatGPT have expanded this interface from scripted coaching to open-ended counseling, but empirical evaluations highlight an important limitation: fluency does not necessarily ensure accuracy. When Ponzo et al. (106) asked a panel of nutrition experts to evaluate ChatGPT-generated dietary plans for several non-communicable diseases, concordance with clinical guidelines was only moderate, ranging from 55.5% for sarcopenia to 73.3% for non-alcoholic fatty liver disease. In complex multimorbidity cases, the model also produced contradictory or inappropriate recommendations. These findings indicate that current LLMs cannot substitute for dietitians in individualized meal planning. Rather, LLM-generated recommendations should be regarded as decision-support outputs that require professional review, particularly when dietary planning involves multimorbidity, therapeutic nutrient restrictions, food allergies, or culturally specific dietary requirements. In these settings, dietitians remain essential for interpreting AI outputs, assessing their clinical appropriateness, and adapting recommendations to individual nutritional needs. Liao et al. (107) reported a similarly mixed picture when 30 senior dietitians evaluated ChatGPT-generated dietary advice for college students. The model achieved 84.38% on an objective nutrition-literacy test, exceeding the performance of the Taiwanese college-student reference group, yet fully met scenario-specific requirements in only 7.50%-37.56% of cases. Among the 242 feedback items, the most frequent concern, accounting for 52 items, was that the information lacked sufficient comprehensiveness and rigor and could therefore be misused. One potential strategy to address these limitations is a hybrid architecture. Papastratis et al. (108) combined a deep generative model with ChatGPT in a PN recommendation system and evaluated the generated plans against EFSA and WHO nutritional requirements across 3,000 virtual and 1,000 real user profiles. The hybrid system outperformed a ChatGPT-only recommender in nutritional accuracy and interpretability, while an expanded dish database increased meal variety.

Overall, these studies reveal a consistent pattern: strong performance on general nutrition knowledge tasks but persistent limitations in quantitative reasoning and completeness. Unconstrained generative models are therefore not yet suitable for autonomous dietary prescription. Retrieval-augmented generation and knowledge-graph-grounded architectures represent promising strategies for constraining model outputs using curated nutritional knowledge and guideline-based rules (see Section 5.5). However, improved nutritional accuracy or guideline concordance in prototype systems should not be interpreted as evidence of established clinical safety, and prospective safety evaluation remains necessary before these approaches can support autonomous dietary prescription. The evaluation and safety requirements that remain are discussed further in Section 7.6.

### 5.5 KGs and personalized food recommendation systems

Predictive models estimate individual responses, whereas language models facilitate human–computer interaction. KGs serve a different role by providing structured nutritional knowledge that supports verifiable and constraint-aware recommendations. Early AI-driven recipe recommendation systems relied more on behavioral patterns than on semantic knowledge. By analyzing large-scale user interaction data from Allrecipes, Elsweiler et al. (109) identified systematic biases in food choice and showed that incorporating these biases as “nudge” signals into recommendation algorithms could improve the healthiness of recommended recipes, as assessed using the UK Food Standards Agency nutrient profiling model, without substantially compromising recommendation accuracy.

A subsequent line of research focused on constructing explicit semantic resources. Haussmann et al. (110) developed FoodKG, a semantics-driven KG integrating approximately one million recipes from Recipe1M+, nutrient data from the United States Department of Agriculture, and health-related guidelines. The platform supports SPARQL-based constrained question answering and can retrieve recipes that meet user-specific dietary restrictions, including allergies and nutrient requirements. Building on this resource, Chen et al. (111) formulated personalized food recommendations as a constrained question-answering task over a large-scale food KG. Their pFOoDReQ framework integrates user queries, dietary preferences, allergies, and constraints derived from American Diabetes Association guidelines, including negation and numerical comparisons. On a benchmark constructed from FoodKG and ADA guidelines, the framework achieved an average absolute improvement of 59.7% across evaluation metrics compared with non-personalized baselines, while providing healthier and more relevant recipe recommendations. KGs can also support personalization at the ingredient level. Shirai et al. (112) combined FoodKG with word-embedding methods, including Word2Vec and spaCy, while using graph embeddings as a comparative baseline, to identify ingredient substitutions that balance flavor compatibility, nutritional characteristics, and dietary restrictions such as allergies and chronic-disease-related contraindications. This approach enables recipes to retain palatability while satisfying individualized health constraints.

Min et al. (113) reviewed these developments across food science and industry applications and highlighted several persistent challenges, including heterogeneous data sources, limited annotation, the need for dynamic knowledge updating, and insufficient explainability. Grounding large language model outputs in KGs containing verified nutritional information may therefore represent a promising direction for improving the reliability and transparency of personalized food recommendation systems. At present, however, this should be regarded primarily as a proposed technical mitigation strategy rather than a clinically validated safety mechanism. Existing studies have mainly demonstrated improvements in constraint satisfaction, recommendation relevance, or nutritional accuracy, rather than reductions in clinically important dietary errors or adverse outcomes. However, systematic evidence demonstrating that KG-based recommendations translate into sustained changes in actual dietary behavior or improved clinical safety remains limited.

### 5.6 Digital twins and adaptive closed-loop systems for real-time nutritional intervention

The approaches reviewed above largely provide static or episodic outputs, such as predicted responses, dietary assessments, or individualized meal plans. A major emerging direction is the development of closed-loop systems in which continuously collected physiological data are integrated into individualized computational models to generate real-time dietary feedback. A subset of such systems may meet the stricter criteria for digital twins defined below. In this review, we use the term “digital twin” in a stricter sense to refer to a dynamic, individualized computational representation of a real person that is continuously or repeatedly updated using real-world physiological and behavioral data and is used to simulate, predict, or optimize future states or interventions. This concept should be distinguished from broader adaptive digital health systems, which may update recommendations in response to newly collected data but do not necessarily maintain a persistently synchronized individualized computational representation or simulate alternative future states.

CGM represents a prototypical data stream for both digital-twin and adaptive closed-loop approaches. Vettoretti et al. (114) reviewed the combined use of AI and CGM sensors in advanced diabetes management, highlighting the role of ML in short-term glucose prediction, risk stratification, and clinical decision support. High-frequency CGM data provide the temporal information required to train individualized glycemic models and support closed-loop management strategies, but the presence of continuous sensing alone is not sufficient to classify a system as a digital twin.

Clinical evidence from systems described in the original studies as digital-twin platforms is beginning to emerge. In a 1-year RCT, Joshi et al. (115) evaluated a digital-twin-labeled platform that integrated CGM, dietary intake, physical activity, and sleep data to generate real-time PN recommendations for patients with type 2 diabetes. Compared with standard care, the intervention group showed significant improvements in metabolic dysfunction-associated fatty liver disease, including reductions in hepatic steatosis and liver enzyme indices. Shamanna et al. (116) further reported that a similarly digital-twin-labeled personalized nutrition platform was associated with improved glycemic control and a significantly higher rate of type 2 diabetes remission than the control condition (72.7% vs. 0% at 1 year). Importantly, the designation “digital twin” reflects the terminology used in the original reports rather than an independent classification by this review. Under the stricter definition adopted here, such platforms should not be regarded as fully realized digital twins unless they explicitly demonstrate continuous or repeated synchronization of an individualized computational representation with real-world data and use that representation to simulate, predict, or optimize future states or interventions. In the absence of these features, they may be more appropriately interpreted as adaptive closed-loop digital health systems. In addition, the findings reported by Shamanna et al. were derived from an ongoing open-label trial reported by the manufacturer and therefore require cautious interpretation and independent validation. Disease-specific applications are discussed further in Section 6.

A parallel line of research has explored whether reliance on invasive or semi-invasive sensing can be reduced. Huang et al. (117) developed a LightGBM ensemble model using multimodal signals from non-invasive wearable devices, including skin temperature and blood-volume pulse, to predict individualized postprandial interstitial glucose excursions in healthy individuals without requiring dietary records. Across a primary dataset comprising more than 1,550 CGM measurements and a follow-up validation dataset with more than 14,400 measurements, the model achieved a root-mean-square error (RMSE) of 18.49 ± 0.1 mg/dL and a mean absolute percentage error (MAPE) of 15.58 ± 0.09%, providing explicit prediction-error estimates in addition to conventional measures of predictive association. These findings suggest that wearable-derived digital biomarkers may partially complement or substitute for CGM in some settings, potentially reducing the cost and invasiveness of real-time dietary monitoring. Nevertheless, the magnitude and clinical acceptability of such prediction errors should be considered when evaluating the potential use of these models in real-time nutritional or glycemic management.

Overall, the closed-loop framework shifts PN from a one-time prescription toward continuous, data-driven adaptation. However, the supporting evidence remains limited. Digital-twin studies are still relatively few and often based on single research groups, while wearable-based glucose estimation retains prediction errors that may be clinically important, particularly in insulin-dependent populations.

## 6 AI-driven PN in disease prevention and management

Disease prevention and management increasingly require nutritional interventions tailored to individual characteristics, with the goals of reducing disease risk while improving treatment outcomes, disease control, and overall health in patients with established disease. The data available for such personalization have expanded rapidly. Advances in genomics and other multi-omics technologies, together with electronic health records (EHRs) and multidimensional health information encompassing behavioral, socioeconomic, and clinical factors, have accelerated the development of clinical data science. Predictive models based on ML algorithms, including logistic regression, support vector machines, and random forests, can identify patterns in large-scale genomic datasets and translate high-dimensional SNP data into estimates of disease risk, prognosis, and treatment response (118). Complementary bioinformatics tools, such as PolyPhen-2 and MutationTaster, assess the potential pathogenicity of individual missense variants by integrating sequence homology, evolutionary conservation, and structural features (118–120). Together, these approaches provide a computational foundation for the ongoing transition in clinical nutrition from systems that primarily predict individual dietary responses toward closed-loop intervention frameworks that translate prediction into personalized recommendations and actions. Representative studies across these application domains are summarized in Table 1.

**Table 1:** Representative studies of artificial intelligence applications in personalized nutrition.

Study (Year)	Design/*N*	AI approach	Application domain	Key finding
Zeevi et al. (96)	Prospective cohort, *n* = 800 (validation *n* = 100); 46,898 meals	Gradient-boosting regression integrating clinical, dietary, and microbiome data	Postprandial glycemic response (PPGR) prediction	Identical meals elicited highly heterogeneous PPGRs; the model accurately predicted individual PPGRs in an independent cohort, and an algorithm-generated diet lowered postprandial glucose.
Korem et al. (97)	Randomized crossover trial, *n =* 20	ML prediction from baseline gut microbiome	Bread-induced glycemic responses	No average difference between breads, but individual glycemic responses were opposite in direction and predictable from baseline microbiome data alone.
Berry et al. (13)	PREDICT 1 cohort, *n =* 1,002 (US validation *n =* 100)	Gradient-boosting ML on multimodal data	Postprandial metabolic response prediction	Model predicted postprandial glycemic (r = 0.77) and triglyceride (r = 0.47) responses; genetic factors explained less than 10% of glycemic variance.
Ben-Yacov et al. (99)	Six-month RCT, *n =* 225 adults with prediabetes	ML-generated personalized postprandial-targeting (PPT) diet vs. Mediterranean diet	Prediabetes glycemic management	PPT diet reduced daily time above 140 mg/dL (−1.3 vs. −0.3 h/day, *P* < 0.001) and HbA1c (−0.16% vs. −0.08%, *P* = 0.007) more than the Mediterranean diet.
Rein et al. (123)	Randomized crossover pilot (*n =* 23) plus 6-month open intervention (*n =* 16)	ML-based PPT diet	Newly diagnosed type 2 diabetes	Six-month PPT intervention reduced HbA1c by 0.39% and fasting glucose by 16.4 mg/dL; 61% of participants achieved diabetes remission (HbA1c < 6.5%).
Mathioudakis et al. (124)	Phase 3 non-inferiority RCT, *n =* 368	Reinforcement-learning fully automated AI diabetes prevention program vs. human coach	Diabetes prevention	Primary composite endpoint reached by 31.7% (AI) vs 31.9% (human coach), meeting the non-inferiority margin; program initiation was higher with AI (93.4% vs. 82.7%).
Myers et al. (102)	System development; volume-estimation experiments on 11 assembled replica meals (NFood-3d, 1,050 depth images)	Deep convolutional neural networks with depth-based volume estimation	Automated dietary assessment	Im2Calories achieved 79% top-1 food-recognition accuracy on the Food-101 test set; end-to-end calorie estimation was not quantitatively evaluated.
Thames et al. (15)	Benchmark dataset of approximately 5,000 real dishes	Deep learning direct nutrient regression from video	Image-based nutrient estimation	Nutrition5k showed direct nutrient regression from food imagery is feasible, and video-based portion estimation can outperform human visual estimation.
Oh et al. (105)	Systematic review of 9 studies (4 RCTs, 5 quasi-experimental)	AI conversational chatbots	Behavior change (diet, physical activity, weight)	Preliminary evidence suggested chatbots may improve physical activity, but definitive conclusions on efficacy for physical activity, diet, or weight management could not be drawn.
Ponzo et al. (106)	Expert panel evaluation of LLM-generated dietary plans	Large language model (ChatGPT)	Dietary planning for non-communicable diseases	Concordance with clinical guidelines was only moderate, ranging from 55.5% (sarcopenia) to 73.3% (non-alcoholic fatty liver disease), with contradictory advice in multi-morbidity cases.
Haussmann et al. (110)	Knowledge-graph resource (~1 million recipes)	Semantics-driven knowledge graph (FoodKG)	Constraint-aware food recommendation	Integrating Recipe1M+, USDA nutrient data, and health guidelines enabled SPARQL-based retrieval of recipes compatible with allergies and nutrient requirements.
Joshi et al. (115)	1-year RCT in type 2 diabetes	Digital-twin-labeled platform integrating CGM, diet, activity, and sleep data	Real-time personalized nutrition prescription	The intervention arm showed significant improvement in metabolic dysfunction-associated fatty liver disease, with reduced hepatic steatosis and liver-enzyme indices vs. standard care.
Huang et al. (117)	Main study (>1,550 CGM measurements) plus validation (>14,400 measurements)	LightGBM ensemble on non-invasive wearable signals	Non-invasive postprandial glucose prediction	Predicted individualized postprandial glucose excursions without dietary logs, with RMSE of 18.49 ± 0.1 mg/dL and MAPE of 15.58 ± 0.09%.
Yin et al. (129)	INSCOC cohort, *n =* 14,134 patients with cancer	Unsupervised clustering plus multinomial logistic regression	Cancer nutritional risk stratification	Clustering identified one well-nourished (58.0%) and three malnutrition clusters concordant with standard criteria; malnutrition severity was inversely associated with survival.
Chen et al. (130)	Multicenter retrospective cohort, *n =* 3,896 patients with cancer	ML feature ranking with logistic regression	Cachexia and precachexia screening	Logistic regression achieved an AUC of 0.830 for cachexia screening and 0.701 for precachexia diagnosis, with satisfactory calibration and favorable decision-curve performance.

AI, artificial intelligence; AUC, area under the receiver operating characteristic curve; CGM, continuous glucose monitoring; HbA1c, glycated hemoglobin; INSCOC, Investigation on Nutrition Status and Clinical Outcome of Common Cancers; LLM, large language model; MAPE, mean absolute percentage error; ML, machine learning; PPT, personalized postprandial-targeting; RCT, randomized controlled trial; RMSE, root-mean-square error; USDA, United States Department of Agriculture.

This section evaluates how effectively the technologies described in Section 5 perform in clinical settings, with particular attention to RCTs, effect sizes, and translational readiness across major disease domains. The strength of the available evidence varies considerably. Glycemic management in T2DM and prediabetes currently has the most mature trial-based evidence, whereas findings for obesity and behavioral interventions remain mixed. By contrast, applications in CVD, oncology, and gastrointestinal disorders are still supported largely by prototype systems, retrospective modeling studies, and small-scale pilot investigations.

### 6.1 Obesity and weight management

Obesity represents an intuitive target for AI-driven dietary personalization. However, available trial evidence indicates that algorithmic individualization does not necessarily translate into superior weight-loss outcomes. One of the most informative studies to date is the Personal Diet Study, a single-center 6-month RCT in which Popp et al. (121) randomized 204 adults with obesity and abnormal glucose metabolism (mean BMI, 33.9 ± 4.8 kg/m^2^) to either an ML-personalized diet, in which an algorithm predicted individual PPGRs and a mobile application provided color-coded meal scores, or a standardized low-fat diet. Contrary to the study hypothesis, weight loss at 6 months did not differ significantly between groups (−4.31% in the low-fat group vs. −3.26% in the personalized group; between-group difference, 1.05%; 95% confidence interval (CI), −0.40 to 2.50; *P* = 0.16). Thus, targeting ML-predicted glycemic responses alone did not outperform a conventional evidence-based diet for weight reduction. The trial also assessed adherence and self-monitoring, with self-monitoring adherence being higher in the personalized group (54.3% vs. 41.1%); however, whether these factors mediate weight-loss outcomes remains to be determined through dedicated mediation analyses (121).

A complementary strategy focuses on personalizing not dietary content itself, but the timing and delivery of behavioral support. Forman et al. (122) evaluated this approach in a proof-of-concept RCT of a just-in-time adaptive intervention (JITAI). A total of 181 adults with overweight or obesity (mean BMI ≈ 34.3 kg/m^2^) received a commercial digital weight-loss program with or without the OnTrack application, whose ML model predicted imminent risk of dietary lapses and delivered tailored interventions at high-risk moments. Among participants receiving the Beyond the Scale program, those in the OnTrack-augmented group lost 4.7% of body weight at 10 weeks compared with 2.6% in the control group (interaction *P* = 0.002). However, the relationship between the model's predictive accuracy and the magnitude of weight loss remains to be clarified (122). Taken together, these trials suggest that the incremental benefit of AI-based personalization is inconsistent and may depend less on model complexity than on how algorithmic outputs are integrated with the broader intervention context, including self-monitoring, behavioral engagement, feedback delivery, adherence, and the underlying dietary intervention (121, 122). Accordingly, observed differences between intervention groups should not automatically be attributed to the AI algorithm itself.

### 6.2 Type 2 diabetes and glycemic management

Glycemic management currently represents one of the most evidence-rich areas of AI-assisted PN, with support from mechanistic cohort studies, RCTs, and real-world implementation. A consistent finding across studies is that identical meals can elicit highly heterogeneous PPGRs between individuals. ML models integrating glycemic parameters, dietary habits, physical activity, and gut microbiome features can predict these responses with good accuracy, whereas genetic factors appear to explain less than 10% of the variance in glycemic responses (13, 96).

Prediction alone, however, does not establish clinical benefit; the more relevant question is whether interventions based on these predictions improve metabolic outcomes. Ben-Yacov et al. (99) addressed this question by randomizing 225 adults with prediabetes to an AI-enabled personalized postprandial-targeting (PPT) intervention combining ML-based dietary personalization with a CGM-linked application or a Mediterranean diet for 6 months. The personalized intervention produced greater reductions in daily time above 140 mg/dL (−1.3 vs. −0.3 h/day; between-group *P* < 0.001) and HbA1c (−0.16% vs. −0.08%; *P* = 0.007), with these differences maintained through 12 months of follow-up (99). However, because the intervention combined algorithmic personalization with CGM monitoring, app-based feedback, and repeated participant interaction, the observed benefit cannot be attributed exclusively to the AI algorithm itself; rather, the trial evaluated the effectiveness of the integrated AI-enabled intervention package. Similar findings have been reported in newly diagnosed T2DM. In a randomized crossover pilot study involving 23 participants, 2 weeks of a PPT diet significantly improved CGM-derived outcomes compared with a Mediterranean diet, including mean PPGR (difference, −19.8 mg/dL × h), mean glucose (−7.8 mg/dL), and time above 140 mg/dL (−2.42 h/day; all *P* < 0.001). A subsequent 6-month open-label PPT intervention in 16 participants reduced HbA1c by 0.39%, fasting glucose by 16.4 mg/dL, and TGs by 49 mg/dL. In addition, 61% of participants achieved diabetes remission, defined as HbA1c < 6.5%, and metabolic improvements were associated with individual changes in the gut microbiome (123). These studies therefore support the potential clinical value of AI-enabled personalized interventions for glycemic management, while the independent contribution of algorithmic personalization relative to monitoring, feedback, adherence, and other intervention components remains to be established. Compared with the neutral weight-loss findings observed in obesity trials, the overall intervention-level evidence currently appears more favorable when glycemic control, rather than weight reduction, is the primary therapeutic target.

A second line of evidence has examined whether AI-based lifestyle interventions can perform comparably to human coaching. In a phase 3 pragmatic non-inferiority RCT, Mathioudakis et al. (124) randomized 368 adults with prediabetes and overweight or obesity to either a fully automated AI-powered Diabetes Prevention Program (DPP), in which a reinforcement-learning algorithm delivered personalized push notifications through an application and Bluetooth-connected scale, or a remotely delivered human-coached DPP for 12 months. The primary composite endpoint was achieved by 31.7% of participants in the AI group and 31.9% in the human-coached group (risk difference, −0.2%; one-sided 95% CI lower bound, −8.2%), meeting the prespecified −15% non-inferiority margin. Program initiation was also higher in the AI group (93.4% vs. 82.7%) (124). These findings support the feasibility of fully automated AI-based lifestyle interventions as a scalable alternative to human-delivered diabetes prevention programs. Real-world evidence provides additional support for this approach. Among 3,148 users of an AI-powered app-based DPP, mean weight loss at the lowest recorded weight was 4.2%, and 35% of users achieved at least 5% weight loss. Participants who met Centers for Disease Control and Prevention curriculum requirements lost 5.3% of body weight at 12 months, compared with 3.3% among non-completers (*P* = 0.015). Logistic regression further identified the number of days of AI-coach interaction (β = 0.43) and self-weighing at least twice weekly (β = 0.68) as significant predictors of clinically meaningful weight loss (125). These findings further indicate that user engagement is likely an important determinant of the effectiveness of AI-supported digital diabetes prevention programs.

### 6.3 CVD and hypertension

Given the central role of diet in cardiovascular prevention, one might expect relatively mature clinical evidence for AI-assisted PN in this area. However, the available evidence remains limited and is still dominated by engineering prototypes and single-arm studies rather than controlled trials. One of the most clinically advanced reports is a 24-week single-arm study by Leitner et al. (126), in which 141 adults with hypertension used a fully autonomous digital platform that collected lifestyle and blood pressure data, developed personalized ML models to predict each user's blood pressure, and autonomously delivered lifestyle recommendations consistent with evidence-based clinical guidelines. The results were encouraging: systolic/diastolic blood pressure decreased by 5.6/3.8 mmHg at 12 weeks and by 8.1/5.1 mmHg at 24 weeks. Among participants with stage 2 hypertension at baseline, the 24-week reduction reached 14.2/8.1 mmHg, while the proportion achieving blood pressure control increased by 26.5%. Engagement was 92%, and only 5.9% of participants required human intervention (126). Nevertheless, the absence of a control group limits causal inference. Earlier, Sookrah et al. (127) described an ML-based Dietary Approaches to Stop Hypertension (DASH) recommendation system using decision-tree (J48) and collaborative-filtering approaches to generate personalized meal suggestions based on demographic, clinical, and preference profiles. Although the system demonstrated high technical accuracy, it has not yet been validated for clinical outcomes. Cardiovascular nutrition therefore illustrates a recurring gap between technical development and clinical validation: evidence-based dietary frameworks such as DASH are increasingly being incorporated into AI systems, whereas randomized evidence demonstrating patient benefit remains limited.

### 6.4 Cancer nutrition and supportive care

Malnutrition and cachexia are common in patients with cancer, have important prognostic implications, and frequently remain underrecognized, making them potential targets for automated nutritional risk assessment. In a systematic review, Sguanci et al. (128) concluded that AI could support automated malnutrition screening, imaging-based body composition assessment, including DL-based identification of skeletal muscle on computed tomography, and PN intervention. However, most available models still lack external validation and prospective evidence of clinical benefit (128). One of the largest studies to date analyzed data from 14,134 Chinese patients with cancer enrolled in the Investigation on Nutrition Status and Clinical Outcome of Common Cancers (INSCOC) cohort. Unsupervised clustering identified a well-nourished cluster comprising 58.0% of participants and three additional clusters characterized by mild, moderate, and severe malnutrition. These clusters showed moderate concordance with the Patient-Generated Subjective Global Assessment and Global Leadership Initiative on Malnutrition criteria. Increasing malnutrition severity was progressively associated with poorer survival, and multinomial logistic regression was the best-performing algorithm, achieving near-perfect malnutrition grading in the validation set (129). For cancer cachexia specifically, a multicenter retrospective cohort study of 3,896 patients with cancer, including 471 with precachexia and 1,178 with cachexia, used ML to identify the most informative clinical features. These included altered food intake, mid-upper-arm circumference, high-density lipoprotein cholesterol, and the C-reactive protein-to-albumin ratio. Logistic regression achieved an area under the receiver operating characteristic curve (AUC) of 0.830 for cachexia screening and 0.701 for precachexia diagnosis, with satisfactory calibration and favorable decision-curve performance, indicating that the study evaluated not only discrimination but also calibration and potential clinical utility (130). Despite these promising results, the available evidence remains predominantly retrospective and diagnostic. Interventional RCTs are still needed to determine whether AI-guided nutritional management can improve oncological outcomes or survival.

### 6.5 Gastrointestinal disorders

Dietary therapy is a mainstay of management for irritable bowel syndrome (IBS) and inflammatory bowel disease (IBD), yet elimination diets are difficult to personalize and often challenging to follow, creating a potential role for AI-assisted tools. The first RCT in this area randomized 58 patients with IBS to either an AI-assisted dietary application (Heali) supporting implementation of a low-fermentable oligosaccharide, disaccharide, monosaccharide, and polyol (FODMAP) diet or conventional dietary education for 4 weeks. In the completer analysis, the application group showed a greater improvement in quality of life (31.1 vs. 11.8 points, *P* = 0.04), a further 24% reduction in total IBS symptom severity that did not reach statistical significance, and approximately twice the reduction in dissatisfaction with bowel habits (*P* = 0.05) (131). A 9-week single-arm pilot study further explored personalized elimination diets. Among 37 participants (16 with IBS, 9 with IBS and Crohn's disease, and 12 with IBS and ulcerative colitis), an ML tool identified each individual's three to five strongest food triggers and generated a personalized elimination diet accordingly. At mid-intervention, 81% of participants reported significant symptom improvement, 70% maintained improvement at the endpoint, 67.6% achieved complete symptom resolution, and 89% reported improved quality of life, with daily engagement of 95% and adherence of 89% (132). However, the absence of a control group limits interpretation of these findings. A third study shifted from symptom-guided to mechanism-guided personalization. In 37 children with active Crohn's disease, ML was used to predict fecal calprotectin responses to exclusive enteral nutrition (EEN). Responders (15/37) had lower baseline fecal butyrate, acetate, and phenylacetate levels and higher microbial richness, while a multi-omic model combining fecal metabolites, dietary data, and microbiota effectively predicted non-response to EEN (133). Although these findings are encouraging, the available studies remain small (*n* = 37–58), and adequately powered RCTs are needed before AI-personalized dietary therapy can be routinely integrated into gastrointestinal practice.

### 6.6 Microbiota-targeted interventions: probiotics and beyond

Probiotics are non-pathogenic microorganisms with defined strain identities that may modulate host physiology through effects on microbial metabolism, pathogen colonization, and host–microbe interactions (134–137). Strain-specific antimicrobial activity and broader health effects have also been reported for Lactobacillus and other lactic acid bacteria (138–140). More recently, microbiota-targeted strategies have expanded beyond conventional probiotics toward next-generation strains selected on the basis of disease-associated microbial and functional signatures (141). Probiotics are non-pathogenic microorganisms with defined strain identities that may modulate host physiology through effects on microbial metabolism, pathogen colonization, and host–microbe interactions (134–137). More recently, microbiota-targeted strategies have expanded beyond conventional probiotics toward next-generation strains selected on the basis of disease-associated microbial and functional signatures (141). From the perspective of AI-assisted PN, the principal relevance of these interventions lies not in the general mechanisms of probiotic action, but in the possibility of using baseline microbiome and multi-omic features to predict which individuals are most likely to respond to a specific microbiota-targeted intervention.

Interventional evidence remains limited but is beginning to emerge. In a randomized, double-blind, placebo-controlled multicenter trial involving healthy older adults, 12 weeks of oral supplementation with probiotics containing *Bifidobacterium bifidum* BGN4 and *Bifidobacterium longum* BORI improved cognitive flexibility and reduced psychological stress, with microbiome changes assessed using 16S ribosomal RNA gene sequencing and bioinformatics analysis (142). Beyond probiotic supplementation itself, AI may contribute at two levels. First, ML models incorporating baseline microbiome features can help predict individual responses to specific interventions. The glycemic-response models discussed above provide a representative example, in which microbiome composition contributed to PPGR prediction and metabolic improvements were associated with individual microbiome changes (96, 123). Second, multi-omic ML frameworks can relate intervention response to microbial signatures, as demonstrated by the prediction of EEN response in pediatric Crohn's disease using fecal metabolomic and microbiota profiles (133). If validated prospectively, these approaches could support more individualized selection of probiotic strains, prebiotic combinations, and accompanying dietary interventions. However, prospective trials evaluating AI-guided probiotic selection have not yet been conducted.

### 6.7 Mobile health and AI-driven behavior change

The effectiveness of PN interventions ultimately depends on sustained patient adherence, making mobile health (mHealth) platforms—particularly conversational AI—an important delivery layer for the approaches described above. The nine-study systematic review discussed in Section 5.4 similarly reached cautious conclusions regarding the effectiveness of behavior-change chatbots (105). A complementary conceptual framework, the AI Chatbot Behavior Change Model, identifies four key components: designing chatbot characteristics according to user backgrounds, building relational capacity, developing persuasive conversational capacity, and evaluating behavioral mechanisms and outcomes. Social cognitive theory was among the behavioral theories incorporated into the studies underpinning this framework (143). In a 12-week single-arm study involving 81 participants, an IBM Watson-powered virtual health coach (“Paola”) delivered personalized guidance on physical activity and the Mediterranean diet. Participants increased physical activity by 109.8 min/week, improved their Mediterranean diet score from 3.8 to 9.6 (+5.7 points), and reduced body weight by 1.3 kg and waist circumference by 2.1 cm. Retention reached 90%, and no adverse events were reported (144).

However, findings from larger controlled studies have been less encouraging. In a multifactorial RCT embedded in the Danish National Birth Cohort, Bjerregaard et al. (145) randomized 7,890 adolescents to an SMS-based chatbot delivering a healthy-eating intervention. In the intention-to-treat analysis, the intervention had no significant effect on dietary habits or weight trajectories. Per-protocol analysis, including the 63% who actively participated, identified only a modest improvement in weight at 6 months, which was no longer evident at 18 months (145). Considered together with the null weight-loss findings reported by Popp et al. (121) in Section 6.1, these results suggest that benefits observed in small, short-term, highly engaged cohorts may diminish substantially when interventions are implemented at population scale. Long-term engagement and adherence therefore appear to be major determinants of effectiveness, beyond the sophistication of the underlying algorithm.

### 6.8 Summary and evidence gaps

When interpreted according to the evidence framework described above, substantial differences emerge between technical performance, clinical efficacy, implementation effectiveness, and long-term patient benefit across disease domains. Three broad conclusions emerge. First, the added value of AI-based personalization over standardized dietary approaches appears to be context dependent. Evidence supports clinical efficacy for several glycemic endpoints (99, 123), remains inconclusive for weight loss (121), and is still insufficient in several other disease areas. Second, automation itself appears feasible, as fully autonomous AI delivery was non-inferior to human coaching in diabetes prevention (124), although implementation effectiveness may be constrained by declining engagement and adherence over time (125, 145). Third, the highest level of evidence—improvement in long-term patient-important outcomes—remains largely absent. Most available RCTs have follow-up periods of 6 to 12 months and focus on intermediate or surrogate outcomes, such as body weight, HbA1c, or CGM-derived glycemic metrics. Among the trials identified in this review, none has demonstrated reductions in incident T2DM, cardiovascular events, or mortality, and at least one large study reported attenuation of intervention effects by 18 months (145). The evidence gap is even greater in oncology and gastroenterology, where retrospective prediction models and small pilot studies are relatively common (129–132), but prospective evidence showing that technical predictive performance translates into clinical efficacy and ultimately patient-important outcomes remains limited. More importantly, future trials should distinguish the effect of algorithmic personalization itself from that of the surrounding digital-intervention package. Dismantling, factorial, or otherwise appropriately controlled trial designs could help separate the contributions of AI-based recommendation algorithms from monitoring intensity, feedback frequency, behavioral support, and adherence.

## 7 Challenges and limitations

The preceding sections have outlined an expanding range of technical approaches and an emerging, although uneven, clinical evidence base for AI-enabled PN. However, a substantial gap remains between technological potential and routine clinical implementation. These systems operate at the intersection of two fields that have historically faced limitations in data quality and infrastructure, while their outputs may directly influence clinical decisions and patient safety. This concern is particularly relevant for populations that are underrepresented in the datasets used for model development. The major barriers to translation include data quality and standardization, algorithmic bias and health equity, explainability and clinical trust, privacy and data governance, regulatory pathways, and risks specific to LLMs. This section examines these challenges and the potential strategies for addressing them. Table 2 summarizes the major limitations, supporting evidence, and candidate mitigation approaches.

**Table 2:** Key challenges of AI-enabled personalized nutrition and potential mitigation strategies.

Challenge	Representative evidence	Potential mitigation/future direction
Data quality and standardization	Self-reported intake is systematically underreported, and bias increases with BMI; nutrient estimates vary across food composition databases	Objective capture via image-based assessment and wearables; harmonized databases under FAIR principles; coordinated data standards
Algorithmic bias and health equity	~78–81% of GWAS participants are of European ancestry; correcting a cost-proxy label bias would raise Black patients receiving additional care from 17.7% to 46.5% (152)	Deliberately inclusive multi-ethnic nutrition–omics cohorts (All of Us model); total-product-lifecycle bias governance; improving digital access
Explainability and clinical trust	*Post hoc* explainability methods can generate misleading “explanations” that manufacture false confidence (155)	Prioritize rigorous prospective clinical validation over *post hoc* rationalization; reserve interpretable models for mechanism-critical settings
Privacy, ethics, and data governance	GDPR classifies genetic data as special-category requiring explicit consent; China's PIPL restricts sensitive health data and cross-border flows	Federated learning across institutions without raw-data exchange; WHO six ethical principles; robust de-identification
Regulation and clinical translation	AI-enabled PN trials show heterogeneous clinical effectiveness: benefits have been reported for glycemic endpoints (99, 123), whereas superiority for weight loss remains inconsistent (121), and long-term effectiveness may be limited by declining engagement (145)	PN-specific prospective validation; CONSORT-AI reporting; monitoring of dynamically updated recommendations; nutrition-specific safety evaluation; and dietitian-led human–AI collaboration
LLM-specific risks	Hallucinated facts and references; guideline concordance of only 55.5%−73.3%; documented errors in allergen meal plans	Proposed/emerging mitigation strategies: retrieval- and knowledge-graph-grounded architectures; prospective hallucination auditing, guideline-concordance testing, adversarial testing, and dietitian verification. Clinical safety benefits remain to be prospectively demonstrated

CONSORT-AI, consolidated standards of reporting trials–artificial intelligence extension; FAIR, findable, accessible, interoperable, reusable; GDPR, General Data Protection Regulation; GWAS, genome-wide association studies; LLM, large language model; PIPL, Personal Information Protection Law; WHO, World Health Organization.

### 7.1 Data quality and standardization

The performance of any predictive model is fundamentally constrained by the quality of its input data, and this issue is particularly pronounced in PN. A major limitation is the reliance on self-reported dietary intake. In a classic methodological analysis, Schoeller (146) demonstrated that energy intake estimated from 24-h recalls and food records systematically differed from energy expenditure measured using doubly labeled water, indicating that self-reported intake does not reliably reflect actual consumption. Subsequent evidence further showed that underreporting is common in both adults and children, increases with BMI, and varies across food types. These biases can attenuate diet–disease associations and limit the validity of self-reported energy intake for energy-balance research in obesity (147). For AI models, this issue is particularly important because biased training labels that are associated with the phenotype of interest may cause models to learn measurement artifacts rather than underlying biological patterns. This limitation provides a rationale for the objective dietary assessment approaches described in Section 5, including image-based methods and wearable sensing, although these technologies also introduce their own sources of measurement error.

Accurate recording of food intake addresses only part of the problem, as the subsequent conversion of dietary records into nutrient exposures introduces additional uncertainty. Ferraz de Arruda et al. (148), in a comparison of fatty-acid data across food composition databases, showed that estimates for the same food can vary substantially depending on the database used. Such differences reflect both systematic and random errors and make database selection itself a source of uncertainty in precision nutrition pipelines. These concerns have contributed to long-standing efforts toward harmonization and standardization, including initiatives such as INFOODS and EuroFIR. A further challenge arises from the integration of multi-omics data. Methodological reviews consistently identify high dimensionality, heterogeneity across omics modalities, and extensive missing data as major barriers to effective integration. Batch-effect correction, data imputation, and cross-modality alignment also remain important methodological challenges, including in advanced deep generative approaches (149). Because the integration of genomic, metabolomic, and microbiome data underpins many of the predictive models discussed in Section 5, limitations at the data-integration stage can propagate directly into PN predictions and recommendations. Addressing these problems will therefore require coordinated data standards and harmonized analytical frameworks rather than improvements in algorithmic complexity alone.

### 7.2 Algorithmic bias and health equity

The equity of an AI model depends heavily on the populations represented in its training data, and the genomic foundation of precision nutrition remains markedly unbalanced. Popejoy and Fullerton (150) estimated that approximately 81% of participants in GWAS were of European ancestry, and by 2018 this proportion had declined only modestly to about 78% (151). Models developed from such datasets often show reduced transferability across populations. In nutrigenomic applications, including polygenic risk scores for glycemic traits, lipid metabolism, and obesity susceptibility, this imbalance may substantially reduce predictive accuracy in Asian, African, and admixed populations. This issue is particularly relevant to PN because nutrition–omics datasets from Asian populations, especially Chinese populations, remain limited. Consequently, models developed predominantly in European or North American cohorts may not generalize well to Chinese dietary patterns, allele frequencies, or gut microbiome profiles.

Algorithmic bias can also arise from model design rather than population representation alone. Obermeyer et al. (152) provided a prominent example by examining a commercial algorithm used to guide healthcare management for millions of patients. At the same algorithmic risk score, Black patients were substantially sicker than White patients because healthcare expenditure had been used as a proxy for health status. Correcting this bias would have increased the proportion of Black patients identified for additional care from 17.7% to 46.5%. This example has direct implications for AI-based nutrition systems: readily available proxy variables, such as purchasing behavior, healthcare utilization, or application engagement, may reflect socioeconomic circumstances rather than underlying physiological status and may therefore introduce systematic bias. Addressing such problems requires governance across the entire model lifecycle rather than isolated performance audits. A consensus framework developed with participation from the United States Food and Drug Administration identifies potential sources and measures of bias throughout the product lifecycle, including data collection, model development, deployment, and post-deployment monitoring, while also recognizing unequal access to healthcare as a potential source of AI-related inequity (153). Similarly, the World Health Organization has cautioned that AI systems trained predominantly on data from high-income populations may perform poorly or even cause harm when applied to more diverse populations in low- and middle-income countries (154).

### 7.3 Explainability and clinical trust

Recommendations that influence patient decisions or override clinician judgment require adequate justification. However, PN faces a persistent challenge in this regard: the models achieving the highest predictive performance are often the least interpretable. *Post hoc* explainability approaches, including saliency maps and feature-attribution methods, have been widely proposed as potential solutions, yet their reliability in healthcare applications remains debated. Ghassemi et al. (155) argued that current explainable AI techniques may generate misleading explanations that create unwarranted confidence in model outputs, suggesting that complex black-box models should be evaluated primarily through rigorous prospective clinical validation rather than *post hoc* interpretation. This concern is particularly relevant in nutrition care, where misleading attribution of dietary or biological factors could distort clinical decision-making. Therefore, rather than pursuing explainability as a universal requirement for all models, the field should prioritize evidence demonstrating that AI-generated recommendations improve outcomes under real-world conditions, while retaining interpretable model classes for scenarios where mechanistic understanding is essential. Explainability remains valuable, but primarily as a complement to empirical validation rather than a replacement for it.

### 7.4 Privacy, ethics and data governance

PN platforms often collect some of the most sensitive categories of health-related information, including genomic profiles, continuous physiological measurements, and detailed dietary behaviors. Such data may reveal not only health status but also cultural practices, religious preferences, mental health characteristics, and socioeconomic conditions. Existing regulatory frameworks have attempted to address these concerns, although their requirements may create challenges for AI development and large-scale data utilization. Under the European Union General Data Protection Regulation (GDPR), genetic information is classified as a special category of personal data under Article 9 and generally requires explicit consent for processing. Meanwhile, principles of data minimization and purpose limitation may conflict with the extensive data reuse required for AI model development, making secondary use dependent on research exemptions and robust de-identification approaches (156). China has established a similarly multifaceted regulatory framework. The Personal Information Protection Law (157) classifies medical and health information as sensitive personal information requiring separate consent procedures and operates alongside the Data Security Law, Cybersecurity Law, and human genetic resources regulations that regulate data management and cross-border transfer. For multinational nutrition–omics collaborations, practical challenges remain regarding informed consent, international data sharing, and regulatory coordination (158, 159). Beyond regulatory compliance, broader governance principles are also essential. The WHO guidance on ethics and governance of AI for health proposes six core principles: protecting autonomy (including consent and privacy), promoting human wellbeing and safety, ensuring transparency and explainability, establishing accountability, promoting inclusiveness and equity, and supporting responsiveness and sustainability (154). Federated learning offers a technical complement, training models across institutions without exchanging raw data and easing the tension between data protection and large, diverse training sets (160).

### 7.5 Regulatory pathways and clinical translation

For AI-enabled PN, regulatory and implementation considerations depend strongly on the intended use of the system and the degree to which its outputs influence clinical dietary decisions. This is particularly relevant for systems that integrate continuously updated CGM, wearable-device, dietary, and behavioral data to generate individualized recommendations, because their outputs may evolve over time rather than remain fixed. In this context, regulatory concerns extend beyond algorithm performance to include the validity of incoming data, the safety of dynamically updated dietary recommendations, transparency of algorithm changes, and responsibility for reviewing and acting on the generated advice.

Broader medical-AI regulatory frameworks provide useful principles for addressing these issues. Gerke et al. (161) proposed a “system view” approach, in which regulators assess the broader human–AI system, including users, clinical environments, and workflow integration, rather than evaluating software in isolation. Similarly, emerging approaches such as predetermined change control plans and post-market real-world performance monitoring may be relevant to AI-enabled PN systems whose algorithms or recommendations are updated after deployment (162). However, these general frameworks require adaptation to nutrition-specific risks and workflows, including repeated dietary modification, integration of wearable and CGM data, and professional oversight of patient-specific recommendations.

Beyond regulatory procedures, a more fundamental challenge is the limited evidence base supporting clinical adoption. This limitation is already evident within AI-enabled PN itself. As discussed in Section 6, randomized studies have yielded heterogeneous results: AI-enabled personalized interventions have shown benefits for glycemic endpoints in some studies (99, 123), whereas superiority for weight loss has not been consistently demonstrated (121), and the effectiveness of large-scale digital nutrition interventions may diminish as engagement declines over time (145). These findings indicate that technical performance or successful personalization does not necessarily translate into sustained clinical effectiveness.

Evidence from other medical AI domains provides a broader cautionary context rather than direct evidence for PN. In a systematic review of DL applications in medical imaging, Nagendran et al. (163) identified substantial limitations in prospective and real-world validation. These findings reinforce the need for PN-specific prospective evaluation that considers not only predictive accuracy but also dietary safety, adherence, sustainability, and clinically meaningful outcomes. Improved reporting standards may partially address this problem. The CONSORT-AI extension introduces 14 AI-specific reporting items, including algorithm version, input and output characteristics, human–AI interactions, and error-case management, to enhance transparency and reproducibility (164).

Clinical translation also depends on workforce adaptation rather than technology alone. Digital tools are increasingly influencing every stage of the nutrition care process, including assessment, diagnosis, intervention, and monitoring. Dietitians therefore have a central role in the implementation of AI-enabled PN, including validating input data, interpreting algorithmic outputs, translating predictions into practical dietary interventions, monitoring adherence and nutritional adequacy, and identifying recommendations that may be inappropriate for complex clinical conditions. Dietitians are therefore encouraged to actively participate in this transformation by developing digital competencies rather than being displaced by automated systems (165). In the near term, the most practical and defensible approach is likely to be a dietitian-led human–AI collaboration model, in which AI supports data integration, risk prediction, and recommendation generation, while dietitians and other qualified healthcare professionals retain responsibility for clinical interpretation, management of complex cases, and final dietary decision-making.

### 7.6 Risks specific to large language models

Beyond the opportunities discussed in Section 5, LLMs introduce unique risks that differ from those associated with conventional predictive models and therefore require dedicated governance strategies. The most prominent concern is hallucination, referring to the generation of plausible but inaccurate information or fabricated references, together with additional challenges related to training-data bias, limited causal reasoning, privacy protection, and accountability (166). In PN, these risks have direct clinical implications. When asked to generate 56 hypothetical meal plans for individuals with food allergies, ChatGPT produced generally acceptable recommendations but demonstrated the potential to generate unsafe dietary advice, with errors particularly related to portion sizes, caloric estimation, and insufficient allergen management (167). Similarly, expert evaluations of ChatGPT-generated dietary recommendations for non-communicable diseases revealed only moderate concordance with clinical guidelines and identified contradictory recommendations in complex multimorbidity scenarios, leading authors to conclude that current LLMs cannot replace healthcare professionals [(106); details in Section 5.4]. Therefore, LLMs in PN are best positioned as supervised tools for information support, drafting, and patient engagement rather than autonomous systems for dietary prescription. Although retrieval augmentation, KG grounding, and other constrained-generation approaches may reduce some classes of factual or guideline-related errors, their ability to improve clinical safety has not yet been established prospectively. Effective governance will therefore require prospective safety evaluation, including hallucination assessment, guideline-concordance evaluation, adversarial testing, clinically relevant error analysis, and dietitian verification, particularly under high-risk scenarios such as elimination diets, food allergies, and complex multimorbidity. These evaluations should demonstrate not only improved model accuracy but also a reduction in clinically meaningful unsafe recommendations before autonomous clinical use is considered. These measures should be integrated with the broader regulatory and reporting frameworks described above to establish reliable implementation pathways.

Collectively, these challenges indicate that the major barriers facing AI-enabled PN are increasingly related to infrastructure, clinical evidence, and ethical governance rather than algorithmic capability alone. Addressing biased and heterogeneous data sources, strengthening prospective evidence, and developing adaptive regulatory frameworks will be essential for translating technological advances into safe and effective clinical practice. These limitations do not diminish the potential of AI in PN; rather, they define the conditions required for its responsible and sustainable implementation.

## 8 Conclusion and future perspectives

This review has traced the development of AI-enabled PN interventions from their biological foundations to emerging clinical applications. The central challenge arises from substantial interindividual variability: genetic variants in loci such as FTO, APOE, and MTHFR influence nutrient metabolism and disease susceptibility; metabolomics provides objective biomarkers of dietary exposure and early metabolic alterations; and the gut microbiota serves as a bidirectional interface linking dietary patterns with metabolite profiles and clinical outcomes. Because no single biological layer can fully explain individual responses, AI provides a framework for integrating diverse data sources. ML models combine clinical, dietary, and microbiome features to predict individual postprandial responses, with reported correlations reaching r = 0.77 (13). DL and CV enable automated dietary assessment, NLP and large language models facilitate nutrition counseling, KGs support constraint-aware recommendations, and digital-twin and adaptive closed-loop approaches integrate continuous monitoring, prediction, and personalized intervention.

Where does the field currently stand? The evidence supports cautious optimism. AI-based PN has progressed beyond proof-of-concept studies toward clinical evaluation, with the strongest evidence emerging from glycemic management in individuals with T2DM and prediabetes [(99, 123, 124); see Sections 6.2 and 6.8]. However, evidence across other applications remains considerably less mature. AI-based personalization has not consistently improved weight-loss outcomes compared with standard dietary approaches (121), chatbot-based interventions have shown reduced effectiveness when scaled to broader populations (145), and applications in CVD, oncology, and gastrointestinal disorders remain largely supported by prototypes and small-scale pilot studies. Two fundamental limitations contribute to this uneven translation. First, the genomic and multi-omics datasets underlying many AI models remain disproportionately derived from individuals of European ancestry (151). Second, the transition from accurate prediction of biological responses to sustained improvements in clinically meaningful outcomes remains largely unresolved.

Addressing these limitations will require coordinated advances across four major areas. First, privacy-preserving computational approaches will be essential for enabling large-scale, multi-center collaboration. Federated learning allows AI models to be trained across institutions and jurisdictions without direct exchange of raw data, offering a potential solution to the tension between data protection requirements, including the GDPR and China's Personal Information Protection Law, and the need for large, diverse datasets (160). Second, intentionally inclusive nutrition–omics cohorts are urgently needed. Initiatives such as the All of Us Research Program, which aims to recruit one million participants with deliberate inclusion of populations historically underrepresented in biomedical research while integrating genomic, EHRs, wearable, and lifestyle data, provide a potential model for developing more representative dietary–metabolomic–microbiome resources, including those relevant to Asian and Chinese populations (168). Third, clinical deployment should prioritize dietitian-led human–AI collaboration rather than professional replacement. In this model, AI functions as a decision-support tool integrated into the nutrition care pathway, while dietitians retain responsibility for translating algorithmic predictions into clinically appropriate dietary interventions, addressing multimorbidity and cultural context, monitoring nutritional adequacy and adherence, and providing final clinical verification. Successful implementation will require systematic development of digital competencies among nutrition professionals (165). Fourth, LLM-based nutrition applications require comprehensive lifecycle safety governance. This should include domain-specific evaluation frameworks incorporating hallucination assessment, guideline-concordance testing, and adversarial evaluation of safety-critical scenarios, together with regulatory approaches for continuously learning systems, including predetermined change control plans, real-world performance monitoring, and AI-specific clinical trial reporting standards (161, 162, 166). The future of PN will not be determined by algorithmic performance alone. If advances in data infrastructure, inclusivity, clinical integration, and safety governance progress together, AI-enabled nutrition may evolve from accurate prediction toward equitable, evidence-based clinical practice. Without these foundations, increasingly sophisticated algorithms alone will be insufficient to overcome the current translational gap.
